# Trends and projections in cutaneous melanoma death in the Netherlands from 1950 to 2045

**DOI:** 10.1097/MD.0000000000027784

**Published:** 2021-12-03

**Authors:** Catharina C. van Niekerk, Hans M.M. Groenewoud, André L.M. Verbeek

**Affiliations:** Radboud Institute for Health Sciences, Department for Health Evidence, Radboud University Medical Center, Nijmegen, The Netherlands.

**Keywords:** age–period–cohort, gender, melanoma, mortality, time trend

## Abstract

Child sun protection has recently been linked to the future disappearance of fatal melanoma in adults in successive generations. In the Netherlands, however, mortality rates from melanoma have increased gradually from the 1950s, with some indication of stabilisation since 2010, which may be compatible with a birth cohort effect by sun-protective measures and screening. To study the trajectories ahead a trend analysis was applied. Numbers of people with cutaneous melanoma as underlying cause of death from 1950 to 2018 and population data were derived from Statistics Netherlands. A graphical approach was used to explore trends in mortality by age, calendar period, and cohorts born in the successive periods of 1889 to 1979. Age–period–cohort modelling outcomes and population forecasts provided projections of mortality until 2045. Based on 24,151 cases of melanoma death (13,256 men, 10,895 women), age-standardised mortality rates were similar from 1950 to 1989 for both genders, and increased thereafter more in men. The age-curve patterns changed gradually towards higher death rates at older age, implying the existence of a birth cohort effect. The age–period–cohort models showed an increase in melanoma mortality rates in successive generations. For women, the birth cohort effect plateaued for generations born since the mid-1980s. The projected total mortality number was predicted to rise in the next 3 decades.

It is concluded that a small future decline of mortality in younger generations can be expected in the Netherlands, but mortality is still rising for the total population.

**Highlights**

- Nearly 1 in 50 cancer deaths in the Netherlands is due to cutaneous melanoma.
- We report a steady increase of age-standardized mortality from the 1950s until very recently.
- Notwithstanding protective measures and screening practice, successive generations born from 1880 to 1980 – the latter birth cohort now reaching the age of 40+ – show increasing melanoma mortality rates.
- A small decline in mortality is observed in younger generations, but in the next 2 decades we will still see rising numbers of melanoma death.

## 1 Introduction

In Europe in 2018, melanoma accounted for 3.7% of the total number of diagnosed cancer cases and 1.4% of the 1.9 million cancer deaths.^[1]^ The estimated age-standardised mortality rate (ASR, European standard population) in Europe in 2018 was 3.2 per 100,000 person–years in men, and 1.9 in women. In comparison, the maximum ASR rate was in Norway with 6.3 and 4.1, and the lowest in Albania 0.8 and 0.6., respectively. The Netherlands rank quite high on this list with 4.6 and 3.1; nearly 1 in 50 cancer deaths are due to cutaneous melanoma.^[2]^

Worldwide, the incidence of melanoma is increasing, while the associated mortality remains stable or is slightly decreasing.^[3]^ Assessing and interpreting the diverging directions of the incidence and mortality rates is complex. In general, time trend mortality alteration mostly reflects changes in early detection and disease treatment.^[4]^ Upward changes in incidence and risk factor prevalence also occur; these have bearing on population mortality, i.e. an increase, whereas more protective factors lead to a mortality decrease. A clear decline in mortality among fair-skinned populations worldwide is suggested in the near future due to reductions in UV-exposure in recent decades, compared to the excessive UV-exposure noted in children for health reasons between 1900 and 1960.^[4–6]^

For populations under observation for several decades, mortality rates are usually classified by calendar year and age at death. If levels of mortality curves change and show a decrease or increase with calendar time, and curves also have different shapes, then an analysis of year-of-birth and age-at-death is appropriate.^[7]^ Nelemans et al,^[8]^ evaluating upward trends in the Netherlands between 1950 and 1988, concluded that time period effects increased up to 1970, most probably due to improved death certification. They also observed an increase in birth cohort effects for cohorts born between 1900 and 1955. For generations born after 1955, mortality appeared to decline. Autier et al^[4]^ stated that cohort effects better explained changes in melanoma mortality over time than period effects. Lifetime risks of death from melanoma increased in successive generations from 1875 onwards and peaked around 1950, whereafter melanoma death gradually decreased in successive generations. The pertinent decline Autier et al predicted after 2040 is questioned by Cayuela et al^[9]^ analysis of Spanish melanoma mortality trends. They found a decrease in the age groups aged under 50, a levelling off in those aged 50 to 59, and an increase in those aged >69.

By studying long-time trends in cutaneous melanoma mortality over 7 decades in a homogeneous population of men and women in the Netherlands, we aim to disentangle the effects of age at death, calendar period (1950–2018), and year of birth (1889–1979), thereby making projections until 2045 possible.

## 2 Material and methods

### 2.1 Mortality and population data

Age and gender-specific data on the number of melanoma deaths and population were obtained from Statistics Netherlands.^[10]^ Data were available from 1950 to 2018, and are presented in 5-year age and period categories (see Tables S1 for men and S2 for women, Supplemental Digital Content).

### 2.2 Statistical methods

#### 2.2.1 Age standardization and joinpoint modelling

We first presented absolute and relative numbers from the 1950s onwards. We then studied time trends of mortality rates per 100,000 person–years after age-standardisation using the direct method and the Netherlands population (2018) as standard. We calculated and graphically presented the numbers and rates over time using Microsoft Office Excel 2010.

Time changes in the age-standardised rates were further examined for the age categories <50, 50 to 69 and 70+ years with the Joinpoint Regression Program, version 4.7 (USA).^[11]^ Joinpoints (moments at which time a significant change in the annual mortality rates occurred) were determined by modelling regression lines before and after the joinpoints. In addition, we calculated the estimated annual percent change (EAPC) of the rates in the time segments.

#### 2.2.2 Age–period–cohort modelling

After descriptive data analysis, we performed age–period–cohort (APC) modelling to quantitatively describe the changing melanoma mortality rates. APC models are Poisson regression models where observed mortality counts are explained by age, period, and cohort categories, based on the assumption that age-specific mortality numbers follow a Poisson distribution.^[12]^ Because there is a linear relation between these 3 variables (if age and period category are known, cohort simply can be computed) a special parameterization in the model is necessary to avoid maximal multicollinearity. All analyses were performed using R [version 3.6.2 (R Foundation, Vienna, Austria)], in particular the Package APC version 1.3.^[13]^

#### 2.2.3 Projections of future burden

The coefficients of the APC regression models were used to extrapolate melanoma mortality rates for periods and generations ahead through to 2045.^[14]^ Predictions on population size and age structure were obtained from Statistics Netherlands.^[10]^ To develop more realistic projections, we adapted the model's coefficients to estimate future mortality, assuming that changes occur gradually. We used a power function to level off the growth, with a projection of the recent linear trend for the last 10 years attenuated by 25%, 50%, and 75% in the second, third, and fourth prediction periods, respectively.

#### 2.2.4 Ethical approval

This study was based on the number of deaths by skin melanoma cancer and publicly available population sizes without personal records, therefore ethical approval and informed consent from participants was unnecessary.

## 3 Results

### 3.1 Time trend patterns

**Figure 1:**
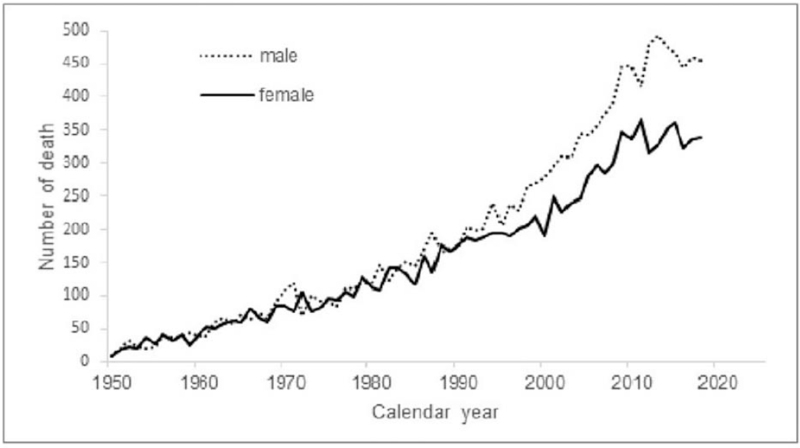
Number of deaths from melanoma in the Netherlands between 1950 and 2018 in men and women.

Since the 1950s, death from melanoma has risen sharply in the Netherlands. Figure 1 shows an increase in deaths in men, rising from 10 to 444 over 7 decades, while in women, we see the same upward trend but at a lower rate from the mid-1990s, with absolute numbers of 10 in 1950 and 338 in 2018.

**Figure 2:**
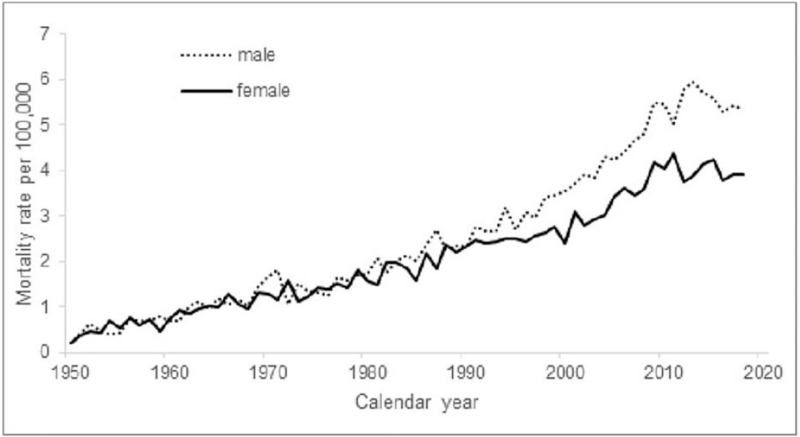
Mortality rates of cutaneous melanoma per 100,000 population in the Netherlands in 1950–2018 by gender.

The burden of melanoma death related to the nation's population is presented in Figure 2 for the period 1950 to 2018. The death rate per 100,000 person–years increases 5-fold for men and nearly quadruples for women.

**Figure 3:**
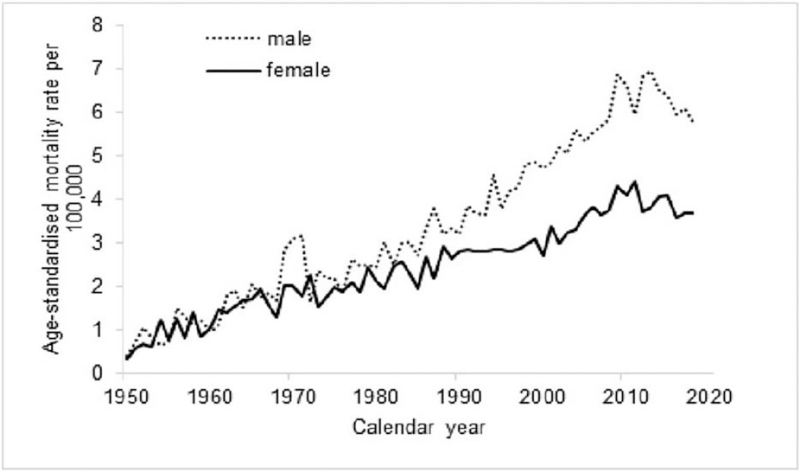
Age-standardised (NL population, 2018) mortality rates of cutaneous melanoma per 100,000 person–years in the Netherlands in 1950–2018 by gender.

The population of the Netherlands has increased from 10 million in 1950 to 17.2 million in 2018. In 1950, nearly 2.5% of the population was aged 70 or over; this is currently 7.0%. To adjust for population size and age composition over time, age-standardised rates were calculated with 2018 serving as standard population, and displayed in Figure 3. Mortality rates increased 12-fold for men; for women 7-fold. From 2000 onwards, the relative risk of melanoma death for men compared to women is 1.7.

**Figure 4:**
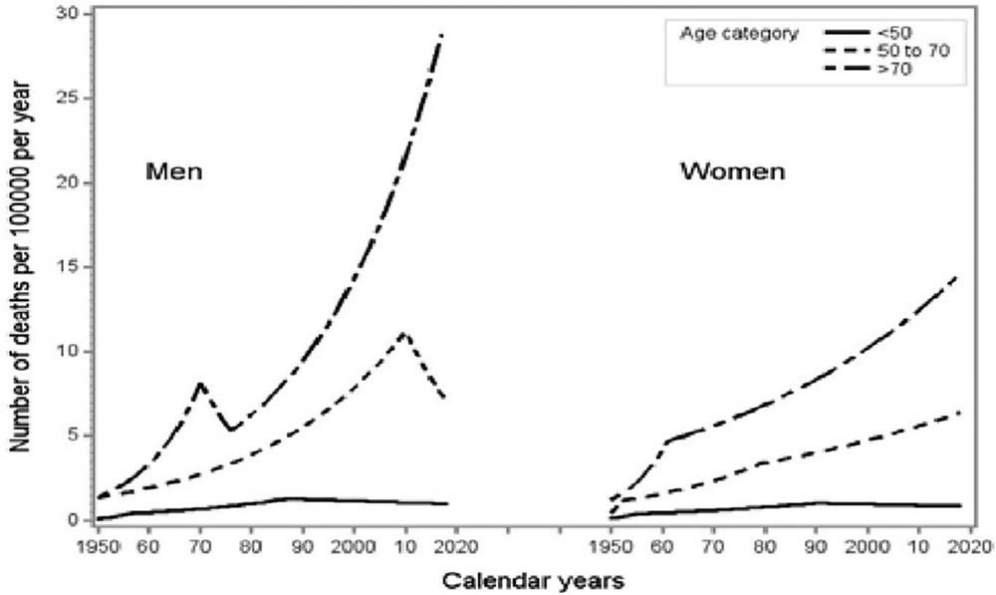
Time trends in melanoma mortality in the Netherlands 1950–2018 analysed by joinpoint models. Adjusted rates are for men and women aged <50, 50–69, and 70+ years.

The results of joinpoint analysis of the age-adjusted rates show low rates for people aged under 50, higher levels for the age group 50 to 69, and double this for those aged 70 and over; Figure 4. For men and women under age 50 the joinpoint models estimated an annual 0.8% increase initially and 0.7% decrease of the rates since the late 1990s. For the male age group 50 to 69, EAPC is 5.6% from 2010 onwards; for females EAPC continues to rise with 1.7% from 1979. The older age groups (70+) still show steadily increasing rates with EAPCs of 5.6% since 1976 for men, and 2.0% since 1961 for women.

### 3.2 Age–period–birth cohort analysis

**Figure 5:**
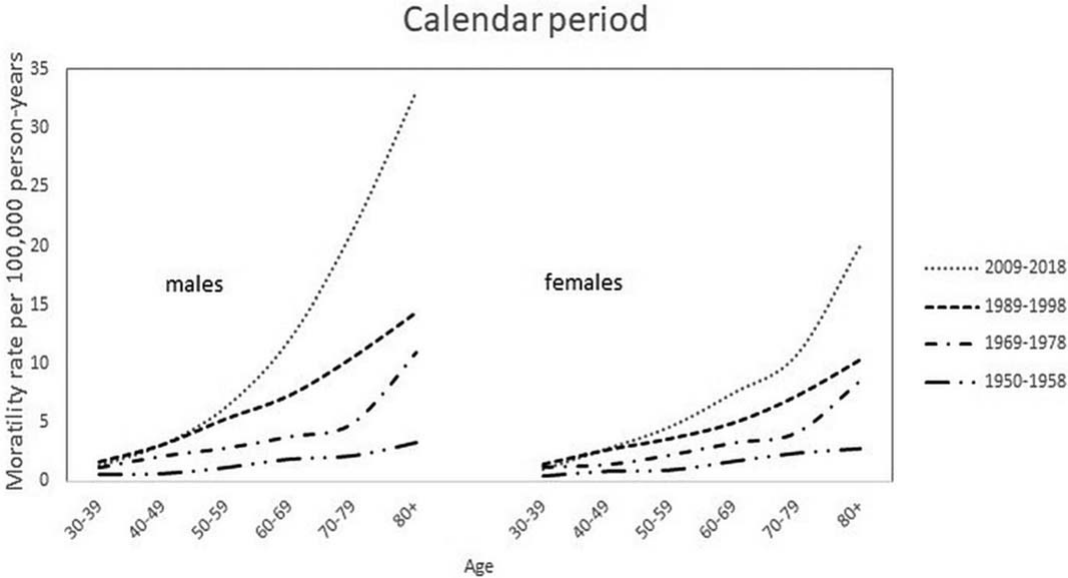
Mortality curves of cutaneous melanoma per 100,000 person–years by age and 10-year calendar periods from 1950–2018 in men and women.

Figure 5 shows age-specific mortality rates for the period 1950 to 2018. The level of the curves continues to rise from the 1950s to well beyond the turn of the century. The shape of the period-specific curves also changes, with increasing rates in the elderly.

**Figure 6:**
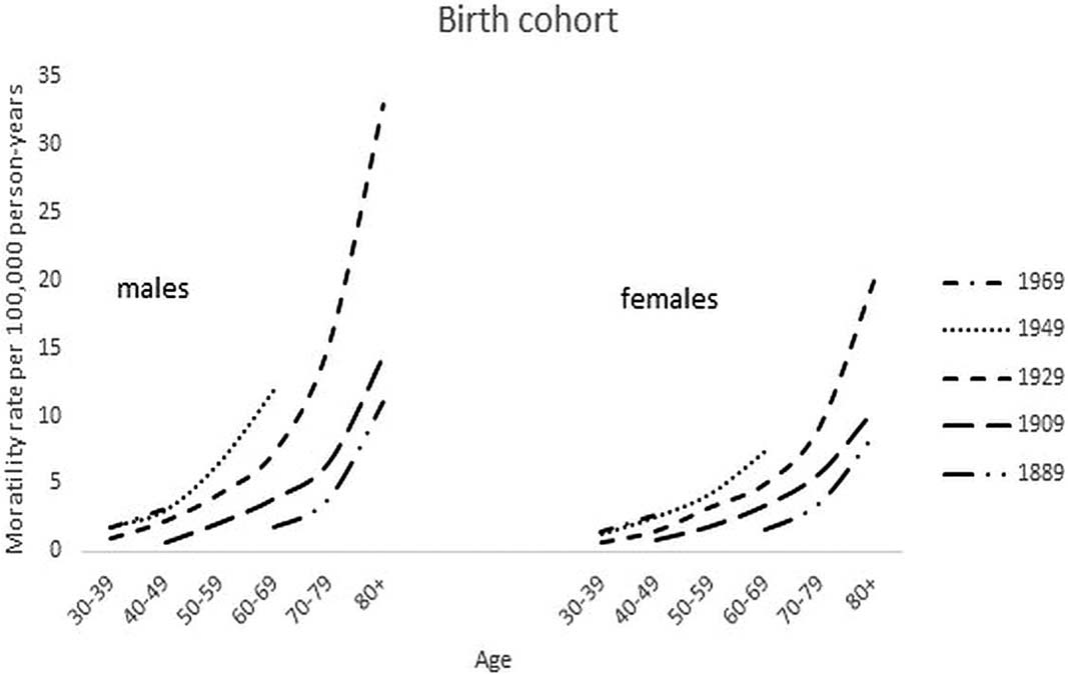
Mortality curves of cutaneous melanoma per 100,000 person–years by age and birth cohort of men and women born in 1889–1979.

The rates according to birth cohort are shown in Figure 6. A consistent age-pattern is observed in both the male and female population: generations born across the continuum of 1889 to 1979 have increasingly higher mortality rates.

**Figure 7:**
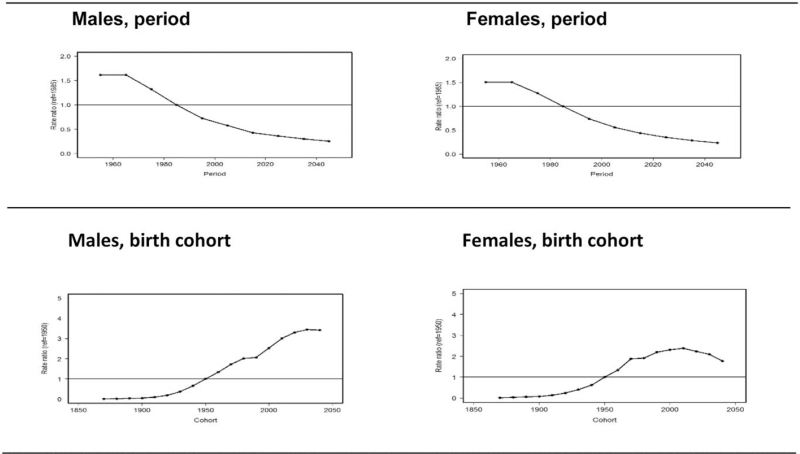
Gender-specific predicted trends of age-adjusted melanoma mortality rate in the Netherlands between 1950 and 2018 and projected trends for the period to 2045 relative to the central year 1980 and findings for birth cohorts from 1870 onwards relative to the 1950 birth cohort.

In order to graphically present the APC models, we transformed the parameter estimates of the calendar period effects and birth cohort effects into rate ratios with the calendar period 1980 to 1989 and the birth cohort 1950 (central year) as reference (rate ratio = 1.0). In this study, they are approximately the median calendar period and birth cohort. The results are presented in Figure 7.

The calendar period effect does not appear to be highly relevant for both men and women (Y-axes). For men, the birth cohort effect shows a relatively stable increasing pattern in risk of death from cutaneous melanoma until recently. For women, the birth cohort effect plateaus for those generations born since the mid-1980s.

### 3.3 Future trajectories

Projections were made on the future predicted population composition. For men, it appears that the occurrence rate has risen from 7.3 per 100,000 population in 2015 to 18.8 in 2045; in women it nearly doubled from 4.5 to 8.1 in the period 2015 to 2045 (Table 1).

**Table 1:** Future numbers of melanoma death and mortality rates in the Netherlands in 2025–2045.

Calendar year	Men	Women	Total
2015	462; 5.55^∗^	339; 3.99	801; 4.76
2025	731; 8.19	446; 4.98	1177; 6.58
2035	1071; 11.51	589; 6.35	1660; 8.94
2045	1453; 15.38	755; 8.05	2208; 14.89

## 4 Discussion

Our major finding was that in the Netherlands over the last 7 decades, age-standardised mortality rates of cutaneous melanoma OSCC increased in men from 0.39 per 100,000 person–years in 1950 to 5.79 in 2018, and in women from 0.34 to 3.66. The changing levels and shape of the age and gender-specific mortality curves prompted us to apply age–period–birth cohort analysis. The results indicated that time period effects decreased slowly from the 1960s onwards. For successive male birth cohorts, mortality from melanoma is still on the rise, whereas for women it is plateauing and gradually decreasing. The projected absolute number of death is predicted to increase steadily in the next 3 decades, from 801 in 2015 to 2208 in 2045.

Our findings are compatible with the rates in Spain, where mortality trends over 1967 to 1986 showed a considerable increase across all age-groups.^[5]^ The Spanish situation in 1975 to 2004 revealed a declining mortality in the younger age-groups and in the more recent birth cohorts, but data from the period 1975 to 2016 did not support this overall decline.^[9,15]^

Autier et al (2015)^[4]^ studied WHO mortality data from countries with mainly light-skinned people, and found that globally, melanoma mortality was still rising in the 2000s. But after these peak years, melanoma deaths in northern Europe (Denmark, Finland, Iceland, Norway and Sweden), were concentrated in older age groups. Also, an abrupt reversal of trends in life-time risks was observed both in men and women, gradually decreasing in successive generations born around 1940.^[4]^ Birth cohort effects showed that mortality would ultimately fade away after 2040 to 2050. They further argued that the temporary epidemic of fatal melanoma was most likely due to the excessive UV-exposure of children that prevailed in 1900 to 1960. Thereafter, mortality decreases would be due to progressive reductions in UV-exposure of children.^[16]^ It should be noted, however, that trend studies using APC-analyses are complex, especially if based on relatively short periods of follow-up and small numbers (stratified to gender).

The strength of our trend study of a homogeneous population is the long follow-up period across 7 decades. A consistent rising pattern of age-standardised rates emerged with either no or just a slight recent decrease. In the 1950s, the small number of cases of <50 a year may be due to underreporting.^[8]^ Between 1980 and 2018, the annual number of melanoma deaths in the Netherlands quadrupled from nearly 200 to over 900. Furthermore, due to the lengthy observation period, a clear gender divide became manifest. These numbers were sufficiently robust to allow Poisson regression for partitioning the influence of age, calendar period, and birth cohort on mortality rate, and to make projections possible.

Investigating this period of 7 decades, we found a gradual increase of melanoma death in both genders that became more prominent in men from the mid-90s. This was repeated with the population-age findings (Figs. 2 and 3), and accumulated into a 60% higher age-adjusted mortality rate in men compared to women after 2010. Age-specific rates across the calendar periods show that the increase was especially evident from age 60 and older for both genders. After rearranging the data for birth cohort display, the shape of the cohort curves were similar in both men and women, but the increase across the successive cohorts appeared to be greater in men (Fig. 7).

It is difficult to find a clear explanation for this gradual divide between men and women. It cannot be specifically attributed to particular age groups, nor to certain birth cohorts. Kölmel et al^[17]^ studied survival probabilities and hazard rates of malignant melanoma in Germany 1972 to 1996, and analysed the evolution of gender differences. They observed that survival of men was lower compared to women and explained their findings by different categories of tumour thickness among the genders. Melanoma awareness campaigns and local screening programmes conducted in Germany since the late 1980s would have resulted in a trend towards a remarkable increase of thin tumours in women compared with men. Similar findings on survival differences between men and women were reported from population-based studies from Sweden, England, and Wales.^[17,18]^

To evaluate diverging directions and sizes of incidence and mortality, homogeneous study populations over several decades of observation are required. According to Netherlands Statistics, the Dutch population is not only increasing and aging in the coming years, but is also becoming more international. More inhabitants will have foreign roots. They are born abroad or have emigrated parents, having different disease rates. At the end of 2019, the Netherlands had 13.2 million inhabitants with a Dutch background and 4.1 million (24%) with a migration background, of whom 85% were European. The number of asylum migrants in 2019 was estimated at 16,000, almost 6% of the total immigration population that year.^[19]^ By 2045, the Dutch population is expected to have grown to 19 million, with around 60% having a nonWestern migrant background. We assume that they will have a lower underlying melanoma incidence rate, making the proportional increase of mortality even more striking.

Additionally, we acknowledge that our study was on routinely collected mortality data derived from Statistics Netherlands, which did not comprise the underlying cause of death by histological subtype of melanoma. Therefore, we were not able to analyse mortality trends in sun-exposure related melanomas vs deaths caused by tumours without known etiological associations with sun exposure. It would have been of importance to see how mortality caused by these 2 distinct types of melanoma is changing over time and could corroborate the expected decline of cutaneous melanoma mortality in light-skinned populations.^[4]^

For the US, Guy et al^[20]^ provided mortality data from the National Center for Health Statistics for the period 1982 to 2011. Melanoma incidence rates increased while mortality rates remained constant. Despite increases in melanoma incidence, decreases in melanoma mortality among those aged <65 years were observed, likely reflecting earlier detection practice and improved treatment. Treatment improvements are expected to affect patients from all ages equally. The authors noted that the national skin cancer prevention programmes should reduce the projected number of melanoma cases over the next 15 years [2015–2030]. The first evidence of effective screening has come from the pilot skin cancer screening project conducted in Schleswig-Holstein, Germany, which achieved a significant 40% melanoma mortality reduction.^[21]^

In all discussions and debates about cancer screening not only the benefit is of importance but also the topic of overdiagnosis. Screening tests also detect tumours that need not cause concern, but nevertheless results in interventions that are not actually necessary. To quantify the problem at the individual and population level is methodologically cumbersome, and which are explanations for the trends in diagnoses.^[22]^

The other influencer of mortality trends is improved treatment, recently obtained with newly developed specific monoclonal antibodies. Among patients with advanced melanoma, the rate of overall survival after 3 years was 58% in the nivolumab-plus-ipilimumab group and 52% in the nivolumab group, compared with 34% in the ipilimumab group; the median overall survival duration has not yet been reached.^[23]^ However, the impact of improvements in the subgroup of patients with advanced cancer will not yet soon become apparent in the total mortality figures. To achieve a decline in melanoma mortality, the emphasis in the forthcoming years will remain on prevention and early diagnosis.^[24]^

## 5 Conclusions

With a 7-decade observation period of cutaneous melanoma death, this study provides insights into the rising mortality rates in men and women. Upward trends are particularly prominent in the 50+ age-group in both genders. A small decline of mortality is observed in younger generations. Notwithstanding effective protective measures and screening practice, mortality from melanoma will continue to increase in the next 2 to 3 decades.

## Acknowledgments

The authors thank Erik Brummelkamp from the Department for Health Evidence from Radboud University Medical Center for scrutinizing the data and for research assistance.

## Author contributions

CCvN, JMMG, and ALMV designed the study. CCvN and JMMG collected study data. Data analysis and interpretation was performed by CCvN, JMMG, and ALMV. The manuscript was drafted by CCvN and ALMV, and CCvN, JMMG, and ALMV critically appraised the manuscript. All authors agreed with submission of the final version of the article.

**Conceptualization:** André L.M. Verbeek.

**Formal analysis:** Hans M.M. Groenewoud.

**Investigation:** Catharina C. van Niekerk.

**Methodology:** Catharina C. van Niekerk, André L.M. Verbeek.

**Software:** Hans M.M. Groenewoud.

**Writing – original draft:** Catharina C. van Niekerk, André L.M. Verbeek.

**Writing – review & editing:** Catharina C. van Niekerk, Hans M.M. Groenewoud.

## Supplementary Material

Supplemental Digital Content
