# Supplementary material for: Trends and projections in cutaneous melanoma death in the Netherlands from 1950 to 2045
Source: Medicine (Baltimore). 2021 Dec 3;100(48):e27784. doi: 10.1097/MD.0000000000027784 (PMC9191606; doi:10.1097/MD.0000000000027784)
Supplement: Supplemental Digital Content [file medi-100-e27784-s001.doc]

| Table S1. Age- and period-specific melanoma mortality and population data for men in the Netherlands 1950─2018 and predicted population size 2015─2045.  S1-1 | | | | | | | | | | | | | | | | | | | | | | | | | | | | | | | | | | | | | | | |  |
| --- | --- | --- | --- | --- | --- | --- | --- | --- | --- | --- | --- | --- | --- | --- | --- | --- | --- | --- | --- | --- | --- | --- | --- | --- | --- | --- | --- | --- | --- | --- | --- | --- | --- | --- | --- | --- | --- | --- | --- | --- |
|  |  |  |  |  |  |  |  |  |  |  |  |  |  |  |  |  |  |  |  |  |  |  |  |  |  |  |  |  |  |  |  |  |  |  |  |  |  |  |  |  |

Men Number of melanoma death

Age yr

| Period | 0-4 | 5-9 | 10-14 | 15-19 | 20-24 | 25-29 | 30-34 | 35-39 | 40-44 | 45-49 | 50-54 | 55-59 | 60-64 | 65-69 | 70-74 | 75-79 | 80-84 | 85-89 | 90-94 | 95+ | Total |
| --- | --- | --- | --- | --- | --- | --- | --- | --- | --- | --- | --- | --- | --- | --- | --- | --- | --- | --- | --- | --- | --- |
| 1950-1953 | 0 | 0 | 0 | 0 | 1 | 2 | 4 | 5 | 5 | 6 | 12 | 10 | 12 | 17 | 6 | 2 | 5 | 1 | 0 | 0 | 88 |
| 1954-1958 | 0 | 0 | 0 | 2 | 3 | 9 | 14 | 11 | 11 | 12 | 11 | 18 | 18 | 12 | 18 | 12 | 6 | 2 | 1 | 1 | 161 |
| 1959-1963 | 1 | 0 | 2 | 2 | 6 | 10 | 13 | 25 | 20 | 17 | 21 | 29 | 21 | 26 | 20 | 13 | 17 | 9 | 0 | 0 | 252 |
| 1964-1968 | 0 | 1 | 0 | 4 | 8 | 9 | 14 | 20 | 22 | 21 | 35 | 38 | 36 | 39 | 35 | 27 | 10 | 8 | 4 | 0 | 331 |
| 1969-1973 | 1 | 0 | 0 | 7 | 9 | 22 | 27 | 28 | 40 | 37 | 46 | 47 | 59 | 33 | 37 | 32 | 32 | 18 | 8 | 6 | 489 |
| 1974-1978 | 0 | 0 | 1 | 3 | 7 | 19 | 28 | 26 | 46 | 45 | 40 | 54 | 54 | 46 | 35 | 37 | 24 | 14 | 6 | 3 | 488 |
| 1979-1983 | 1 | 0 | 0 | 5 | 8 | 28 | 43 | 54 | 51 | 36 | 70 | 87 | 66 | 67 | 44 | 51 | 13 | 19 | 5 | 2 | 650 |
| 1984-1988 | 0 | 0 | 1 | 3 | 13 | 28 | 47 | 76 | 76 | 81 | 71 | 76 | 89 | 76 | 61 | 56 | 44 | 19 | 9 | 0 | 826 |
| 1989-1993 | 0 | 0 | 1 | 5 | 5 | 27 | 44 | 63 | 86 | 89 | 95 | 106 | 101 | 90 | 80 | 74 | 49 | 21 | 7 | 4 | 947 |
| 1994-1998 | 0 | 2 | 1 | 2 | 22 | 16 | 47 | 53 | 79 | 99 | 128 | 106 | 125 | 136 | 145 | 96 | 65 | 37 | 11 | 7 | 1177 |
| 1999-2003 | 0 | 0 | 0 | 3 | 7 | 29 | 46 | 53 | 106 | 105 | 155 | 181 | 165 | 156 | 148 | 150 | 90 | 50 | 15 | 3 | 1462 |
| 2004-2008 | 0 | 0 | 0 | 2 | 7 | 15 | 42 | 77 | 96 | 126 | 141 | 247 | 245 | 241 | 218 | 163 | 114 | 55 | 17 | 2 | 1808 |
| 2009-2013 | 0 | 0 | 1 | 1 | 3 | 17 | 33 | 60 | 106 | 128 | 197 | 216 | 301 | 331 | 302 | 240 | 185 | 98 | 44 | 14 | 2277 |
| 2014-2018 | 1 | 0 | 1 | 3 | 3 | 16 | 22 | 26 | 63 | 96 | 135 | 204 | 248 | 308 | 357 | 324 | 253 | 165 | 61 | 14 | 2300 |

S1-2

| Men |  |  |  |  |  |  |  |  |  |  |  |  |  |  |  |  |  |  |  |  |  |
| --- | --- | --- | --- | --- | --- | --- | --- | --- | --- | --- | --- | --- | --- | --- | --- | --- | --- | --- | --- | --- | --- |
| Population | |  |  |  |  |  |  |  |  |  |  |  |  |  |  |  |  |  |  |  |  |
|  |  |  |  |  |  |  |  |  |  |  |  |  |  |  |  |  |  |  |  |  |  |
|  | Age yr |  |  |  |  |  |  |  |  |  |  |  |  |  |  |  |  |  |  |  |  |
|  |  |  |  |  |  |  |  |  |  |  |  |  |  |  |  |  |  |  |  |  |  |
| Period | 0-4 | 5-9 | 10-14 | 15-19 | 20-24 | 2 5-29 | 30-34 | 35-39 | 40-44 | 45-49 | 50-54 | 55-59 | 60-64 | 65-69 | 70-74 | 75-79 | 80-84 | 85-89 | 90-94 | 95+ | Total |
| 1950-1953 | 2426603 | 2054892 | 1708155 | 1642484 | 1621682 | 1562197 | 1400707 | 1346362 | 1276393 | 1175360 | 1027778 | 895549 | 746039 | 605602 | 455316 | 287279 | 136053 | 47356 | 9018 | 990 | 20425815 |
| 1954-1958 | 2862426 | 2972951 | 2474881 | 2102456 | 2001723 | 1926624 | 1885577 | 1701650 | 1642690 | 1551793 | 1413484 | 1220423 | 1031995 | 830212 | 625080 | 414043 | 209899 | 71297 | 15339 | 1718 | 26956261 |
| 1959-1963 | 2976512 | 2851970 | 2966418 | 2463898 | 2074632 | 1953322 | 1899655 | 1869209 | 1685224 | 1620109 | 1519376 | 1357485 | 1136185 | 922027 | 696426 | 463794 | 250920 | 92139 | 19390 | 2409 | 28821100 |
| 1964-1968 | 3096956 | 2966951 | 2851990 | 2965551 | 2466224 | 2107121 | 1978126 | 1909930 | 1861328 | 1663971 | 1580582 | 1449720 | 1252596 | 999543 | 758840 | 514140 | 282896 | 112718 | 26832 | 3250 | 30849265 |
| 1969-1973 | 3019637 | 3098355 | 2975706 | 2859429 | 2979334 | 2511819 | 2139921 | 1992188 | 1899219 | 1832939 | 1618591 | 1505019 | 1332458 | 1093072 | 808755 | 548803 | 313576 | 129762 | 34056 | 4839 | 32697478 |
| 1974-1978 | 2575023 | 3059310 | 3138029 | 3014343 | 2903012 | 3038248 | 2533885 | 2143172 | 1974284 | 1868121 | 1785474 | 1542535 | 1386608 | 1160392 | 881154 | 579650 | 332217 | 145701 | 41203 | 6986 | 34109347 |
| 1979-1983 | 2268813 | 2625832 | 3106646 | 3195804 | 3077472 | 2958856 | 3048880 | 2519515 | 2118465 | 1939901 | 1818378 | 1706579 | 1425858 | 1214609 | 937643 | 633980 | 353925 | 160390 | 50161 | 10653 | 35172360 |
| 1984-1988 | 2247250 | 2284303 | 2641773 | 3132028 | 3234380 | 3107547 | 2956191 | 3022699 | 2487863 | 2077814 | 1888332 | 1739746 | 1583858 | 1258142 | 992918 | 678735 | 388154 | 169150 | 54219 | 11142 | 35956244 |
| 1989-1993 | 2412538 | 2282772 | 2317142 | 2691144 | 3214666 | 3325498 | 3146468 | 2958726 | 3004016 | 2452645 | 2022463 | 1807087 | 1621004 | 1410983 | 1040843 | 731869 | 417425 | 184605 | 56220 | 12149 | 37110263 |
| 1994-1998 | 2503910 | 2455048 | 2325073 | 2369173 | 2761719 | 3303062 | 3362490 | 3147333 | 2940768 | 2966163 | 2395804 | 1946281 | 1694538 | 1456147 | 1183121 | 777668 | 456048 | 198126 | 58889 | 11174 | 38312535 |
| 1999-2003 | 2557673 | 2540291 | 2496376 | 2398766 | 2441364 | 2844402 | 3338730 | 3360597 | 3127179 | 2904479 | 2901945 | 2314697 | 1839006 | 1535932 | 1239525 | 903083 | 497491 | 220530 | 63080 | 11735 | 39536881 |
| 2004-2008 | 2523738 | 2552586 | 2548146 | 2528289 | 2450099 | 2493399 | 2841244 | 3294979 | 3308176 | 3070080 | 2833821 | 2802671 | 2196097 | 1689964 | 1335198 | 978661 | 602921 | 253792 | 73420 | 12606 | 40389887 |
| 2009-2013 | 2359754 | 2515310 | 2552132 | 2569247 | 2603745 | 2530514 | 2519167 | 2821527 | 3250455 | 3253632 | 3004208 | 2747437 | 2675762 | 2041527 | 1508054 | 1098980 | 696149 | 335246 | 94587 | 16131 | 41193564 |
| 2014-2018 | 2256006 | 2382290 | 2539521 | 2610617 | 2706242 | 2705052 | 2574842 | 2530777 | 2811739 | 3219183 | 3200254 | 2929464 | 2640693 | 2510755 | 1849096 | 1280827 | 816585 | 408068 | 132601 | 21909 | 42126521 |
|  |  |  |  |  |  |  |  |  |  |  |  |  |  |  |  |  |  |  |  |  |  |
|  |  |  |  |  |  |  |  |  |  |  |  |  |  |  |  |  |  |  |  |  |  |
|  |  |  |  |  |  |  |  |  |  |  |  |  |  |  |  |  |  |  |  |  |  |
| Year | 0-4 | 5-9 | 10-14 | 15-19 | 20-24 | 25-29 | 30-34 | 35-39 | 40-44 | 45-49 | 50-54 | 55-59 | 60-64 | 65-69 | 70-74 | 75-79 | 80-84 | 85-89 | 90-94 | 95+ | Total |
| 2015 | 454728 | 476048 | 515675 | 512283 | 542322 | 529400 | 508503 | 500143 | 586818 | 647318 | 638084 | 575631 | 522826 | 503209 | 346275 | 247171 | 158901 | 77940 | 25678 | 3905 | 8372858 |
| 2025 | 470890 | 451393 | 474559 | 504582 | 574133 | 579402 | 585593 | 553295 | 519557 | 506056 | 583173 | 629438 | 604627 | 525927 | 452968 | 400243 | 232065 | 116923 | 3 9759 | 7659 | 8812242 |
| 2035 | 509827 | 511586 | 486359 | 475018 | 525106 | 558297 | 596789 | 577967 | 572053 | 538358 | 505894 | 489401 | 551674 | 577541 | 532312 | 432999 | 322606 | 209517 | 67787 | 13778 | 9054869 |
| 2045 | 484159 | 508038 | 525503 | 535404 | 537889 | 528797 | 546392 | 557396 | 582920 | 560755 | 554486 | 517559 | 477932 | 451035 | 492007 | 488227 | 398504 | 246420 | 105583 | 28309 | 9127315 |
|  |  |  |  |  |  |  |  |  |  |  |  |  |  |  |  |  |  |  |  |  |  |
| Predictions of population size and age structure were obtained from Statistics Netherlands [10]. | | | | | | | | | |  |  |  |  |  |  |  |  |  |  |  |  |

S1-3

Men

| Melanoma mortality rate per 100,000 PY | | | | | |  |  |  |  |  |  |  |  |  |  |  |  |  |  |  |
| --- | --- | --- | --- | --- | --- | --- | --- | --- | --- | --- | --- | --- | --- | --- | --- | --- | --- | --- | --- | --- |
|  | | | | | |  |  |  |  |  |  |  |  |  |  |  |  |  |  |  |
|  | Age yr |  |  |  |  |  |  |  |  |  |  |  |  |  |  |  |  |  |  |  |
| Period | 0-4 | 5--9 | 10-14 | 15-19 | 20-24 | 25-29 | 30-34 | 35-39 | 40-44 | 45-49 | 50-54 | 55-59 | 60-64 | 65-69 | 70-74 | 75-79 | 80-84 | 85-89 | 90-94 | 95+ |
| 1950-1953 | 0.00 | 0.00 | 0.00 | 0.00 | 0.06 | 0.13 | 0.29 | 0.37 | 0.39 | 0.51 | 1.17 | 1.12 | 1.61 | 2.81 | 1.32 | 0.70 | 3.68 | 2.11 | 0.00 | 0.00 |
| 1954-1958 | 0.00 | 0.00 | 0.00 | 0.10 | 0.15 | 0.47 | 0.74 | 0.65 | 0.67 | 0.77 | 0.78 | 1.47 | 1.74 | 1.45 | 2.88 | 2.90 | 2.86 | 2.81 | 6.52 | 58.21 |
| 1959-1963 | 0.03 | 0.00 | 0.07 | 0.08 | 0.29 | 0.51 | 0.68 | 1.34 | 1.19 | 1.05 | 1.38 | 2.14 | 1.85 | 2.82 | 2.87 | 2.80 | 6.78 | 9.77 | 0.00 | 0.00 |
| 1964-1968 | 0.00 | 0.03 | 0.00 | 0.13 | 0.32 | 0.43 | 0.71 | 1.05 | 1.18 | 1.26 | 2.21 | 2.62 | 2.87 | 3.90 | 4.61 | 5.25 | 3.53 | 7.10 | 14.91 | 0.00 |
| 1969-1973 | 0.03 | 0.00 | 0.00 | 0.24 | 0.30 | 0.88 | 1.26 | 1.41 | 2.11 | 2.02 | 2.84 | 3.12 | 4.43 | 3.02 | 4.57 | 5.83 | 10.20 | 13.87 | 23.49 | 123.99 |
| 1974-1978 | 0.00 | 0.00 | 0.03 | 0.10 | 0.24 | 0.63 | 1.11 | 1.21 | 2.33 | 2.41 | 2.24 | 3.50 | 3.89 | 3.96 | 3.97 | 6.38 | 7.22 | 9.61 | 14.56 | 42.94 |
| 1979-1983 | 0.04 | 0.00 | 0.00 | 0.16 | 0.26 | 0.95 | 1.41 | 2.14 | 2.41 | 1.86 | 3.85 | 5.10 | 4.63 | 5.52 | 4.69 | 8.04 | 3.67 | 11.85 | 9.97 | 18.77 |
| 1984-1988 | 0.00 | 0.00 | 0.04 | 0.10 | 0.40 | 0.90 | 1.59 | 2.51 | 3.05 | 3.90 | 3.76 | 4.37 | 5.62 | 6.04 | 6.14 | 8.25 | 11.34 | 11.23 | 16.60 | 0.00 |
| 1989-1993 | 0.00 | 0.00 | 0.04 | 0.19 | 0.16 | 0.81 | 1.40 | 2.13 | 2.86 | 3.63 | 4.70 | 5.87 | 6.23 | 6.38 | 7.69 | 10.11 | 11.74 | 11.38 | 12.45 | 32.92 |
| 1994-1998 | 0.00 | 0.08 | 0.04 | 0.08 | 0.80 | 0.48 | 1.40 | 1.68 | 2.69 | 3.34 | 5.34 | 5.45 | 7.38 | 9.34 | 12.26 | 12.34 | 14.25 | 18.67 | 18.68 | 62.65 |
| 1999-2003 | 0.00 | 0.00 | 0.00 | 0.13 | 0.29 | 1.02 | 1.38 | 1.58 | 3.39 | 3.62 | 5.34 | 7.82 | 8.97 | 10.16 | 11.94 | 16.61 | 18.09 | 22.67 | 23.78 | 25.56 |
| 2004-2008 | 0.00 | 0.00 | 0.00 | 0.08 | 0.29 | 0.60 | 1.48 | 2.34 | 2.90 | 4.10 | 4.98 | 8.81 | 11.16 | 14.26 | 16.33 | 16.66 | 18.91 | 21.67 | 23.15 | 15.87 |
| 2009-2013 | 0.00 | 0.00 | 0.04 | 0.04 | 0.12 | 0.67 | 1.31 | 2.13 | 3.26 | 3.93 | 6.56 | 7.86 | 11.25 | 16.21 | 20.03 | 21.84 | 26.57 | 29.23 | 46.52 | 86.79 |
| 2014-2018 | 0.04 | 0.00 | 0.04 | 0.11 | 0.11 | 0.59 | 0.85 | 1.03 | 2.24 | 2.98 | 4.22 | 6.96 | 9.39 | 12.27 | 19.31 | 25.30 | 30.98 | 40.43 | 46.00 | 63.90 |

| Table S2. Age- and period-specific melanoma mortality and population data for women in the Netherlands 1950─2018 and predicted population size 2015─2045. | | | | | | | | | | | | | | | | | | | | | | | | | | | | | | | | | | | | | | | |  |
| --- | --- | --- | --- | --- | --- | --- | --- | --- | --- | --- | --- | --- | --- | --- | --- | --- | --- | --- | --- | --- | --- | --- | --- | --- | --- | --- | --- | --- | --- | --- | --- | --- | --- | --- | --- | --- | --- | --- | --- | --- |
|  |  |  |  |  |  |  |  |  |  |  |  |  |  |  |  |  |  |  |  |  |  |  |  |  |  |  |  |  |  |  |  |  |  |  |  |  |  |  |  |  |

S2-1

Women Number of melanoma death

|  |  |  |  |  |  |  |  |  |  |  |  |  |  |  |  |  |  | |  |  |  |  |  |
| --- | --- | --- | --- | --- | --- | --- | --- | --- | --- | --- | --- | --- | --- | --- | --- | --- | --- | --- | --- | --- | --- | --- | --- |
| Period |  | Age yr |  |  |  |  |  |  |  |  |  |  |  |  |  |  |  | |  |  |  |  |  |
|  |  |  |  |  |  |  |  |  |  |  |  |  |  |  |  |  |  | |  |  |  |  |  |
|  | 0-4 | 5-9 | 10-14 | 15-19 | 20-24 | 25-29 | 30-34 | 35-39 | 40-44 | 45-49 | 50-54 | 55-59 | 60-64 | 65-69 | 70-74 | 75-79 | | 80-84 | | 85-89 | 90-94 | 95+ | Total |
| 1950-1953 | 0 | 0 | 0 | 0 | 3 | 2 | 4 | 7 | 6 | 6 | 9 | 4 | 11 | 7 | 7 | 5 | | 2 | | 1 | 0 | 0 | 74 |
| 1954-1958 | 0 | 0 | 0 | 1 | 6 | 7 | 5 | 10 | 11 | 24 | 12 | 19 | 14 | 25 | 22 | 12 | | 7 | | 2 | 4 | 0 | 181 |
| 1959-1963 | 0 | 0 | 0 | 1 | 4 | 7 | 10 | 15 | 20 | 20 | 26 | 22 | 19 | 17 | 21 | 22 | | 14 | | 7 | 2 | 2 | 229 |
| 1964-1968 | 0 | 0 | 1 | 2 | 7 | 6 | 12 | 11 | 26 | 38 | 29 | 37 | 32 | 28 | 31 | 29 | | 21 | | 14 | 3 | 1 | 328 |
| 1969-1973 | 0 | 1 | 1 | 3 | 6 | 10 | 16 | 29 | 29 | 32 | 37 | 37 | 36 | 44 | 44 | 37 | | 31 | | 22 | 9 | 0 | 424 |
| 1974-1978 | 0 | 0 | 1 | 0 | 7 | 14 | 35 | 26 | 20 | 32 | 34 | 50 | 50 | 65 | 46 | 34 | | 42 | | 19 | 3 | 1 | 479 |
| 1979-1983 | 0 | 0 | 0 | 1 | 8 | 15 | 37 | 33 | 36 | 35 | 57 | 60 | 61 | 78 | 61 | 61 | | 45 | | 31 | 13 | 1 | 633 |
| 1984-1988 | 0 | 0 | 0 | 3 | 7 | 22 | 39 | 41 | 50 | 56 | 57 | 67 | 60 | 61 | 76 | 74 | 51 | | | 35 | 18 | 4 | 721 |
| 1989-1993 | 0 | 1 | 1 | 0 | 11 | 25 | 42 | 38 | 65 | 85 | 59 | 71 | 72 | 89 | 105 | 80 | 76 | | | 53 | 20 | 7 | 900 |
| 1994-1998 | 0 | 1 | 0 | 4 | 7 | 18 | 32 | 61 | 62 | 77 | 89 | 71 | 90 | 93 | 104 | 103 | 78 | | | 61 | 27 | 9 | 987 |
| 1999-2003 | 0 | 0 | 1 | 2 | 5 | 18 | 25 | 65 | 64 | 69 | 89 | 120 | 90 | 104 | 110 | 137 | 99 | | | 81 | 40 | 6 | 1125 |
| 2004-2008 | 1 | 0 | 0 | 0 | 6 | 16 | 31 | 56 | 83 | 86 | 135 | 119 | 131 | 136 | 138 | 152 | 157 | | | 99 | 51 | 12 | 1409 |
| 2009-2013 | 0 | 0 | 0 | 2 | 5 | 18 | 24 | 49 | 87 | 108 | 143 | 139 | 177 | 195 | 151 | 164 | 196 | | | 141 | 71 | 25 | 1695 |
| 2014-2018 | 0 | 0 | 1 | 3 | 3 | 6 | 16 | 20 | 65 | 86 | 130 | 136 | 156 | 220 | 212 | 178 | 187 | | | 171 | 93 | 27 | 1710 |

| S2-2  Women | Population | | |  |  |  |  |  |  |  |  |  |  |  |  |  |  |  |  |  |  |
| --- | --- | --- | --- | --- | --- | --- | --- | --- | --- | --- | --- | --- | --- | --- | --- | --- | --- | --- | --- | --- | --- |
|  | Age yr |  |  |  |  |  |  |  |  |  |  |  |  |  |  |  |  |  |  |  |  |
| Period | 0-4 | 5-sep | okt-14 | 15-19 | 20-24 | 25-29 | 30-34 | 35-39 | 40-44 | 45-49 | 50-54 | 55-59 | 60-64 | 65-69 | 70-74 | 75-79 | 80-84 | 85-89 | 90-94 | 95+ | Total |
| 1950-1953 | 2294984 | 1953465 | 1638860 | 1577399 | 1588066 | 1588934 | 1433978 | 1391134 | 1336420 | 1239353 | 1095479 | 951425 | 790307 | 645489 | 490700 | 317596 | 158764 | 58397 | 12547 | 1915 | 20565212 |
| 1954-1958 | 2710978 | 2819853 | 2358172 | 2020700 | 1940650 | 1926549 | 1940561 | 1750134 | 1708152 | 1636515 | 1502983 | 1317597 | 1117985 | 899579 | 685117 | 459518 | 240897 | 88786 | 21239 | 2961 | 27148926 |
| 1959-1963 | 2836263 | 2707169 | 2821275 | 2358040 | 1997998 | 1900438 | 1908668 | 1927821 | 1741899 | 1694217 | 1615015 | 1469299 | 1264612 | 1040400 | 786911 | 532884 | 293746 | 113510 | 27134 | 4265 | 29041564 |
| 1964-1968 | 2950248 | 2831044 | 2711466 | 2826094 | 2335925 | 1970322 | 1890275 | 1901398 | 1918954 | 1727471 | 1669426 | 1576643 | 1411672 | 1178364 | 916102 | 623869 | 352014 | 144651 | 37215 | 5417 | 30978570 |
| 1969-1973 | 2881186 | 2955365 | 2842330 | 2728004 | 2828881 | 2321355 | 1969163 | 1890689 | 1896964 | 1905766 | 1703590 | 1631487 | 1518297 | 1321251 | 1045327 | 736833 | 421348 | 180357 | 49107 | 7805 | 32835105 |
| 1974-1978 | 2458293 | 2921025 | 2996578 | 2886196 | 2785541 | 2850495 | 2333497 | 1983541 | 1898930 | 1889960 | 1884291 | 1668585 | 1577575 | 1429813 | 1187407 | 857031 | 515433 | 225254 | 65572 | 11695 | 34426712 |
| 1979-1983 | 2164529 | 2510473 | 2966949 | 3051618 | 2965092 | 2829203 | 2863182 | 2342133 | 1993112 | 1891298 | 1867051 | 1846128 | 1614137 | 1492829 | 1299964 | 999713 | 628671 | 300544 | 92635 | 18885 | 35738146 |
| 1984-1988 | 2152078 | 2180524 | 2526762 | 2996302 | 3109871 | 2994748 | 2832263 | 2858369 | 2337058 | 1980939 | 1869235 | 1828449 | 1786070 | 1531731 | 1368059 | 1112992 | 753121 | 379135 | 127617 | 26358 | 36751681 |
| 1989-1993 | 2308927 | 2186100 | 2211286 | 2581870 | 3088739 | 3166162 | 3021602 | 2843256 | 2860208 | 2326907 | 1960155 | 1832580 | 1771134 | 1699218 | 1411241 | 1183346 | 851601 | 462417 | 163106 | 36473 | 37966328 |
| 1994-1998 | 2389651 | 2348063 | 2224457 | 2264077 | 2685505 | 3160885 | 3207625 | 3043824 | 2847759 | 2845797 | 2298609 | 1921933 | 1775316 | 1684711 | 1566418 | 1225797 | 912195 | 525736 | 196093 | 43877 | 39168328 |
| 1999-2003 | 2440313 | 2425715 | 2386960 | 2284444 | 2387844 | 2788185 | 3213923 | 3233104 | 3049101 | 2831478 | 2808816 | 2252687 | 1863985 | 1691236 | 1553925 | 1364641 | 952304 | 567434 | 223915 | 52216 | 40372226 |
| 2004-2008 | 2408864 | 2437538 | 2432329 | 2417203 | 2396652 | 2479787 | 2821994 | 3214234 | 3220482 | 3021862 | 2786909 | 2744825 | 2181694 | 1779647 | 1569699 | 1365074 | 1E+06 | 607325 | 248169 | 61604 | 41271272 |
| 2009-2013 | 2250727 | 2402480 | 2437222 | 2454393 | 2546977 | 2497053 | 2512303 | 2820197 | 3196562 | 3194094 | 2978512 | 2726505 | 2660781 | 2088611 | 1668865 | 1404181 | 1E+06 | 720908 | 288187 | 76123 | 42032934 |
| 2014-2018 | 2147227 | 2270811 | 2425449 | 2488506 | 2626069 | 2649646 | 2547028 | 2535867 | 2829614 | 3190370 | 3165179 | 2926960 | 2651527 | 2551318 | 1964890 | 1509905 | 1E+06 | 762495 | 350114 | 89966 | 42845205 |
|  |  |  |  |  |  |  |  |  |  |  |  |  |  |  |  |  |  |  |  |  |  |
|  | 0-4 | 5-9 | 10-19 | 15-19 | 20-24 | 25-29 | 30-34 | 35-39 | 40-44 | 45-49 | 50-54 | 55-59 | 60-64 | 65-69 | 70-74 | 75-79 | 80-84 | 85-89 | 90-94 | 95+ | Total |
| Year | 433594 | 453607 | 493414 | 488710 | 526710 | 521274 | 504238 | 501857 | 590046 | 638599 | 632100 | 575755 | 523358 | 511223 | 369468 | 295363 | 231591 | 149681 | 70400 | 16880 | 8527868 |
| 2015 |
| 2025 | 448126 | 428845 | 451659 | 477746 | 553597 | 558837 | 568029 | 546577 | 521425 | 512761 | 591050 | 627776 | 608830 | 541526 | 475966 | 439702 | 283524 | 176354 | 81077 | 23081 | 8916488 |
| 2035 | 485025 | 486822 | 462474 | 447833 | 502565 | 535450 | 578848 | 565366 | 568243 | 544767 | 516471 | 501862 | 569545 | 595699 | 564843 | 480526 | 380581 | 281012 | 112839 | 32295 | 9213066 |
| 2045 | 460572 | 483414 | 499546 | 506188 | 514753 | 506329 | 528498 | 545583 | 580698 | 562893 | 560812 | 531732 | 498306 | 478274 | 534493 | 541535 | 472386 | 330544 | 169509 | 60206 | 9366271 |

Predictions of population size and age structure were obtained from Statistics Netherlands [10].

S2-3

| Women | Melanoma mortality rate per 100,000 PY | | | | |  |  |  |  |  |  |  |  |  |  |  |  |  |  |  |
| --- | --- | --- | --- | --- | --- | --- | --- | --- | --- | --- | --- | --- | --- | --- | --- | --- | --- | --- | --- | --- |
|  | Age yr | |  |  |  |  |  |  |  |  |  |  |  |  |  |  |  |  |  |  |
| Period | 0-4 | 5-9 | 10-14 | 15-19 | 20-24 | 25-29 | 30-34 | 35-39 | 40-44 | 45-49 | 50-54 | 55-59 | 60-64 | 65-69 | 70-74 | 75-79 | 80-84 | 85-89 | 90-94 | 95+ |
| 1950-1953 | 0.00 | 0.00 | 0.00 | 0.00 | 0.19 | 0.13 | 0.28 | 0.50 | 0.45 | 0.48 | 0.82 | 0.42 | 1.39 | 1.08 | 1.43 | 1.57 | 1.26 | 1.71 | 0.00 | 0.00 |
| 1954-1958 | 0.00 | 0.00 | 0.00 | 0.05 | 0.31 | 0.36 | 0.26 | 0.57 | 0.64 | 1.47 | 0.80 | 1.44 | 1.25 | 2.78 | 3.21 | 2.61 | 2.91 | 2.25 | 18.83 | 0.00 |
| 1959-1963 | 0.00 | 0.00 | 0.00 | 0.04 | 0.20 | 0.37 | 0.52 | 0.78 | 1.15 | 1.18 | 1.61 | 1.50 | 1.50 | 1.63 | 2.67 | 4.13 | 4.77 | 6.17 | 7.37 | 46.89 |
| 1964-1968 | 0.00 | 0.00 | 0.04 | 0.07 | 0.30 | 0.30 | 0.63 | 0.58 | 1.35 | 2.20 | 1.74 | 2.35 | 2.27 | 2.38 | 3.38 | 4.65 | 5.97 | 9.68 | 8.06 | 18.46 |
| 1969-1973 | 0.00 | 0.03 | 0.04 | 0.11 | 0.21 | 0.43 | 0.81 | 1.53 | 1.53 | 1.68 | 2.17 | 2.27 | 2.37 | 3.33 | 4.21 | 5.02 | 7.36 | 12.20 | 18.33 | 0.00 |
| 1974-1978 | 0.00 | 0.00 | 0.03 | 0.00 | 0.25 | 0.49 | 1.50 | 1.31 | 1.05 | 1.69 | 1.80 | 3.00 | 3.17 | 4.55 | 3.87 | 3.97 | 8.15 | 8.43 | 4.58 | 8.55 |
| 1979-1983 | 0.00 | 0.00 | 0.00 | 0.03 | 0.27 | 0.53 | 1.29 | 1.41 | 1.81 | 1.85 | 3.05 | 3.25 | 3.78 | 5.22 | 4.69 | 6.10 | 7.16 | 10.31 | 14.03 | 5.30 |
| 1984-1988 | 0.00 | 0.00 | 0.00 | 0.10 | 0.23 | 0.73 | 1.38 | 1.43 | 2.14 | 2.83 | 3.05 | 3.66 | 3.36 | 3.98 | 5.56 | 6.65 | 6.77 | 9.23 | 14.10 | 15.18 |
| 1989-1993 | 0.00 | 0.05 | 0.05 | 0.00 | 0.36 | 0.79 | 1.39 | 1.34 | 2.27 | 3.65 | 3.01 | 3.87 | 4.07 | 5.24 | 7.44 | 6.76 | 8.92 | 11.46 | 12.26 | 19.19 |
| 1994-1998 | 0.00 | 0.04 | 0.00 | 0.18 | 0.26 | 0.57 | 1.00 | 2.00 | 2.18 | 2.71 | 3.87 | 3.69 | 5.07 | 5.52 | 6.64 | 8.40 | 8.55 | 11.60 | 13.77 | 20.51 |
| 1999-2003 | 0.00 | 0.00 | 0.04 | 0.09 | 0.21 | 0.65 | 0.78 | 2.01 | 2.10 | 2.44 | 3.17 | 5.33 | 4.83 | 6.15 | 7.08 | 10.04 | 10.40 | 14.27 | 17.86 | 11.49 |
| 2004-2008 | 0.04 | 0.00 | 0.00 | 0.00 | 0.25 | 0.65 | 1.10 | 1.74 | 2.58 | 2.85 | 4.84 | 4.34 | 6.00 | 7.64 | 8.79 | 11.13 | 14.60 | 16.30 | 20.55 | 19.48 |
| 2009-2013 | 0.00 | 0.00 | 0.00 | 0.08 | 0.20 | 0.72 | 0.96 | 1.74 | 2.72 | 3.38 | 4.80 | 5.10 | 6.65 | 9.34 | 9.05 | 11.68 | 17.69 | 19.56 | 24.64 | 32.84 |
| 2014-2018 | 0.00 | 0.00 | 0.04 | 0.12 | 0.11 | 0.23 | 0.63 | 0.79 | 2.30 | 2.70 | 4.11 | 4.65 | 5.88 | 8.62 | 10.79 | 11.79 | 16.09 | 22.43 | 26.56 | 30.01 |
|  |  |  |  |  |  |  |  |  |  |  |  |  |  |  |  |  |  |  |  |  |
